# Broad autism phenotype features of Chinese parents with autistic children and their associations with severity of social impairment in probands

**DOI:** 10.1186/s12888-015-0568-9

**Published:** 2015-07-23

**Authors:** Li-Juan Shi, Jian-Jun Ou, Jing-Bo Gong, Su-Hong Wang, Yuan-Yue Zhou, Fu-Rong Zhu, Xu-Dong Liu, Jing-Ping Zhao, Xue-Rong Luo

**Affiliations:** 1 Mental Health Institute of the Second Xiangya Hospital and Key Laboratory of Psychiatry and Mental Health of Hunan Province, The Central South University, 139 Middle Renmin Road, Changsha, Hunan PR China; 2 Traditional Chinese Medicine University of Hunan, Changsha, Hunan PR China; 3 Department of Neuroscience, The Third Affiliated Hospital of Soochow University, Changzhou, Jiangsu PR China; 4 Hangzhou Seventh People’s Hospital, Hangzhou, PR China; 5 Department of Psychiatry, Queen’s University, 191 Portsmouth Ave, Kingston, ON K7M 8A6 Canada

**Keywords:** Broad autism phenotype, Parent, Autism, Social Adjustment, Chinese

## Abstract

**Background:**

Parents of children with autism have higher rates of broad autism phenotype (BAP) features than parents of typically developing children (TDC) in Western countries. This study was designed to examine the rate of BAP features in parents of children with autism and the relationship between parental BAP and the social impairment of their children in a Chinese sample.

**Methods:**

A total of 299 families with autistic children and 274 families with TDC participated in this study. Parents were assessed using the Broad Autism Phenotype Questionnaire (BAPQ), which includes self-report, informant-report, and best-estimate versions. Children were assessed using the Chinese version of the Social Responsiveness Scale (SRS).

**Results:**

Parents of children with autism were significantly more likely to have BAP features than were parents of TDC; mothers and fathers in families with autistic children had various BAP features. The total scores of the informant and best-estimate BAPQ versions for fathers were significantly associated with their children’s SRS total scores in the autism group, whereas the total scores of the three BAPQ versions for mothers were significantly associated with their children’s SRS total scores in the TDC group. In the autism group, the total SRS scores of children with “BAP present” parents (informant and best-estimate) were higher than the total SRS scores of children with“BAP absent” parents. In the TDC group, the total SRS scores of children with “BAP present” parents were higher than the total SRS scores of children with“BAP absent” parents (best-estimate).

**Conclusions:**

Parents of autistic children were found to have higher rates of BAP than parents of TDC in a sample of Chinese parents. The BAP features of parents are associated with their children’s social functioning in both autism families and TDC families, but the patterns of the associations are different.

## Background

Autism is a neurodevelopmental disorder marked by early-onset impairments in social interaction and communication, and unusually restricted, repetitive behavior and interests [1]. Autism has a strong genetic influence, with heritability estimates exceeding 80 % [2]. However, environmental risks and the gene-environment interplay in the etiology of autism are important for interpreting this heritability, which is never 100 %. Identification of autism susceptibility loci is complex and heterogeneous, with up to 1000 genes being implicated [3, 4]. Many genetic variants that are linked to autism have a high degree of pleiotropy (i.e., one gene affects more than one phenotype). To understand the genetic etiology of autism better, some researchers have turned their attention to the milder expression of autistic traits in parents or siblings of individuals with autism, which are referred to as the broad autism phenotype (BAP).

BAP is a term describing a group of mild, non-pathological social dysfunctions, communication traits, and unusual personality features that can often be found in the relatives of individuals with autism [5, 6]. Thus, BAP is thought to reflect a genetic propensity for the disorder. Studies on BAP would be helpful to identify which traits aggregate in family members and are, therefore, useful for restricting phenotypic heterogeneity to detect vulnerability genes for autism more accurately. Researchers from fields of autism genetics [7, 8] and behavioral genetics [9, 10] have strongly recommended such an approach. Prevalence estimates indicate that 14 % to 50 % of family members of individuals with autism exhibit at least one BAP feature [11–13], and some studies have explored the relationship between parental BAP and child social function. Sasson et al. [14] who classified the parents of autistic probands into “BAP present” and “BAP absent,” found that children of “BAP present” parents had higher scores on the Social Communication Questionnaire (SCQ) than those of “BAP absent” parents. Similarly, Maxwell et al. [15] reported that paternal BAP scores significantly correlated with the Social Responsiveness Scale (SRS) scores of their children with autism spectrum disorder. The SCQ and the SRS are commonly used measures of autism.

Although several Western studies have examined the rate of BAP traits in parents with autistic children [11–13, 15], little research has been conducted on this association in China. Cultural differences between China and Western counties might affect the rate of BAP traits in Chinese parents of children with autism. Since the parenting style and family interaction pattern in China are different from those in Western countries, these differences also might affect the association between parental BAP traits and the child’s social function. Therefore, we designed the present study to explore the rate of BAP in Chinese parents with autistic children, and to explore how parental BAP features are associated with the severity of autism symptoms in probands.

## Methods

### Participants

The research protocol for this study was approved by the ethics committee of the Second Xiangya Hospital, Central South University, China. Written informed consent was obtained from all the parents.

Autism families were recruited from special education schools, which provide interventions for children with autism. The families of typically developing children were randomly recruited from two kindergartens in the same city. The inclusion criteria for parents were their written consent to participate, intact families (biological parents still married and living together), with a child living with both parents. The exclusion criteria for parents were severe medical or genetic conditions, history of psychiatric disorders (e.g., autism, developmental disorder, depression, or schizophrenia) or any hearing/vision impairments (other than prescription glasses). Parents of typically developing children also had to have no history of autism or any related developmental disorders in first-degree relatives.

Children with autism had a previous DSM-IV diagnosis of autism made by a licensed clinician experienced in the assessment and diagnosis of autism. Typically developing children who were reported to have developmental disorders and related medical problems (e.g., epilepsy or head injury) were excluded.

A total of 324 families of children with autism and 354 families of typically developing children were recruited for the study from September 2012 to January 2015. Twenty-five families of autistic children and 56 families of typically developing children were excluded because of missing data. Another 24 families of typically developing children were excluded from all the analyses because the children had excessively high total raw scores on the SRS, since a child with a SRS total raw score exceeding 65 may have social problems [16]. This yielded a final sample of 299 families of autistic children and 274 families of typically developing children (Table 1).

**Table 1:** Demographic Information

	PCA (n = 299)	PTC (n = 274)	*t* /χ^2^	*p* value
	Mean or N (SD or %)	Mean or N (SD or %)		
Age of child, Months	59.60(14.89)	58.63(15.14)	−0.771	*0.441*
Gender of child, N (%)			97.10	<0.001
Boys	258(86.3 %)	131(47.8 %)		
Girls	41(13.7 %)	143(52.2 %)		
Child’s SRS total score	98.93(25.35)	43.23(11.29)	−30.532	<0.001
Age of father, Years	35.07(3.80)	34.97(3.94)	−0.318	0.751
Age of mother, Years	32.88(3.42)	32.60(3.40)	−0.973	0.331
Education of father, N (%)			14.610	0.001
Primary to junior high school	29(9.7 %)	58(21.1 %)		
High school to some college	146(48.9 %)	116(42.4 %)		
Undergraduate or above	124(41.4 %)	100(36.5 %)		
Education of mother, N (%)			19.522	<0.001
Primary to junior high school	43(14.4 %)	81(29.5 %)		
High school to some college	139(46.5 %)	102(37.2 %)		
Undergraduate or above	117(39.1 %)	91(33.3 %)		
Family income (Yuan)			44.487	<0.001
Less than 30,000	57(19.1 %)	35(12.8 %)		
30,000-less than 50,000	93(31.1 %)	41(15 %)		
50,000-less than 70,000	48(16.1 %)	34(12.4 %)		
70,000-less than 100,000	43(14.4 %)	53(19.3 %)		
More than 100,000	58(19.4 %)	111(40.5 %)		

PCA: parents of children with autism; PTC: parents of typically developing children. SRS: Social Responsiveness Scale. Sixty-three children with autism and sixty-three typically developing children who were younger than four 4 years old, were excluded from the analyses of SRS scores. Family income: according to the annual per capita income statistics published by the statistics for Hunan province in 2011.

The children ranged in age from 28 months through 155 months. There was no difference in age between the two groups (autism: M = 59.60 months; typically developing children: M = 58.63 months).

### Measurement of parental BAP

The Broad Autism Phenotype Questionnaire (BAPQ) [17] was used to measure parental BAP features. It is a reliable screening tool for detecting BAP in adults, which assesses particular personality and language characteristics along three dimensions — Aloof personality, Rigid personality, and Pragmatic language. All parents were asked to complete the self-report version of the BAPQ, independently, as well as the informant version about their spouse. The BAPQ was presented to them as “The Personality and Preferences Questionnaire” in order to reduce bias associated with beliefs about autism. The validity of the Chinese version of the BAPQ was verified, with the permission of its author (Professor Joseph Piven: Department of Psychiatry, Neurodevelopmental Disorders Research Center, University of North Carolina), by translating it into Chinese, verifying the Chinese version by translating it back to English, and then comparing that version with the original version. Three sets of scores were obtained for each parent: self-report BAPQ scores, informant-report BAPQ scores, and best-estimate scores (the average of the self-report and informant-report scores). We adopted the cutoff values established by Sasson et al. [13] to reduce false positives and to optimize the diagnostic classification of the BAP.

### Measurement of children’s social impairment

The Chinese version of the Social Responsiveness Scale (SRS) [16] was used to measure the social impairment of the children. The original SRS, which was developed by Constantino and colleagues in 2002 [18, 19], is a 65-item self-report or caregiver-report questionnaire for 4–18 years old children, that uses a 4-point Likert-type scale. The SRS, which was completed by the mothers in this study, can be used as a screening tool, as well as an aid for clinical diagnosis. It has subscale scores for specific symptom domains (Social awareness, Social cognition, Social communication, Social motivation, and Autistic mannerisms), and a total score that serves as an index of the severity of social deficits. A higher total SRS score indicates greater severity of social impairment. The SRS is applicable to all participants – not only those with autism – and it is not affected by intelligence, age, race, or the education level of respondents [18, 20]. The Chinese version of the SRS has fair test–retest reliability (intraclass correlations 0.751–0.852), internal consistency (Cronbach’s alpha 0.944–0.947), and convergent validity (Pearson’s correlations 0.609–0.865) [16].

### Statistical analysis

Data analysis was performed using SPSS version 17. All statistical tests were 2-tailed using 0.05 as the level of statistical significance. Continuous variables are described using means and standard deviations (SD), whereas categorical variables are reported using frequencies and percentages. Between-group comparisons were performed using independent t-tests or analysis of variance (ANOVA) for continuous variables, and the Chi-square (χ2) test or Fisher’s exact probability test for categorical variables.

Multivariate analysis of variance (MANOVA) was performed to compare the BAPQ scores between the two parent groups. In this model, the dependent variables were self-report, informant-report, and best-estimate Aloof personality, Pragmatic language, and Rigidity subscales, and the total scores. The between-subject factors were group (PCA vs. PTC) and sex (father vs. mother).

Parents were then categorized as “BAP present” or “BAP absent,” based on whether the BAPQ self-report, informant-report, and best-estimates for the total score or any subscale scores exceeded the cutoff values reported by Sasson et al. (2013) [13]. Chi-square or Fisher’s tests were conducted to determine whether the frequency of BAP features of parents (i.e. the proportion of “present” cases) differed between families of autistic and typically developing children.

To determine if different BAP features co-occur more frequently in parents of children with autism than in parents of typically developing children, the dichotomized “present” scores on each of the three subscales and the total score were summed to indicate the number of BAP features a parent had: 0, 1, 2, 3, 4 (For example, a “3” indicates that a parent had three distinct BAP features.). The proportion of parents with different numbers of BAP features were compared between the groups.

Linear regression analyses were performed to examine whether the BAPQ total scores of parents were associated with the severity of social impairment (SRS) in their children. Paternal and maternal BAPQ total scores were entered in the regression model with child SRS score as the dependent variable. Independent sample t-tests were performed on the SRS total scores between children whose parents were “BAP present” or “BAP absent,” separately for the autism group and the typical development group.

## Results

Parents’ ages were not significantly different between the autism group and typical development group. The education levels of the parents of children with autism were significantly higher than those of parents of typically developing children, but the family income of parents of children with autism was lower than that of parents of typically developing children (Table 1).

### BAPQ scores

Descriptive data for the self-report, informant-report, and best-estimate BAPQ scores of the mothers and fathers in the PCA and PTC groups are presented in Table 2. MANOVA indicated there was a significant main effect of group on self-report, informant-report, and best-estimate Aloof personality subscales, the Pragmatic language subscales, and their total scores, with the parents of children with autism scoring higher than the PTC parents. There also was a significant main effect of sex on the informant-report Pragmatic language subscale, and the total score, and on the best-estimate Rigidity subscale, with mothers scoring higher than fathers. There was no significant interaction between group and sex on the BAPQ scores (See Table 2). Nor did the scores of fathers and mothers within PCA’s differ significantly on any of three versions of the subscales of the BAPQ (all *p*’s ≥0.05).

**Table 2:** Mean BAPQ scores of the two parental groups

	Fathers		Mothers		*F*^1^(1, 1142)	*F*^2^(1, 1142)	*F*^3^(1, 1142)
	PCA	PTC	PCA	PTC	Main effect	Main effect	Main effect
	Mean (SD)	Mean (SD)	Mean (SD)	Mean (SD)	for sex	for group	for sex × group
Self-report BAPQ							
Aloof personality	2.96(0.82)	2.74(0.74)	2.88(0.74)	2.74(0.66)	0.943	15.684^**^	0.93
Pragmatic language	2.53(0.72)	2.32(0.64)	2.47(0.68)	2.37(0.66)	0.002	15.848^**^	1.831
Rigidity personality	3.01(0.59)	3.01(0.52)	3.04(0.62)	3.08(0.51)	2.002	0.348	0.162
Total score	2.83(0.56)	2.69(0.49)	2.80(0.56)	2.73(0.48)	0.001	11.395^*^	1.398
Informant-report BAPQ							
Aloof personality	2.94(0.86)	2.75(0.75)	2.96(0.77)	2.79(0.71)	0.402	14.904^**^	0.076
Pragmatic language	2.61(0.81)	2.38(0.70)	2.71(0.85)	2.52(0.77)	7.022^*^	20.013^**^	0.32
Rigidity personality	3.16(0.73)	3.11(0.57)	3.28(0.70)	3.25(0.59)	10.975^*^	1.003	0.121
Total score	2.91(0.66)	2.75(0.53)	2.98(0.61)	2.86(0.54)	7.278^*^	16.605^**^	0.254
Best-estimate BAPQ							
Aloof personality	2.95(0.740	2.75(0.65)	2.92(0.65)	2.77(0.59)	0.031	20.27^**^	0.498
Pragmatic language	2.57(0.67)	2.35(0.57)	2.59(0.63)	2.45(0.62)	2.718	24.757^**^	1.186
Rigidity personality	3.09(0.55)	3.06(0.46)	3.16(0.54)	3.16(0.45)	8.593^*^	0.102	0.201
Total score	2.87(0.54)	2.72(0.43)	2.89(0.49)	2.79(0.43)	2.793	18.943^**^	0.917

BAPQ: Broad Autism Phenotype Questionnaire; PCA: parents of children with autism; PTC: parents of typically developing children.*p < 0.05;**p < 0.001.

### BAP features frequency

Parents of children with autism had significantly higher rates of BAP features than the parents of typically developing children, except for the self-report Pragmatic language subscale and the informant-report and best-estimate Rigidity subscales for mothers, and the self-report and best-estimate Rigidity subscales and informant-estimate Aloof personality subscale for fathers (Table 3). Among the parents of children with autism, mothers had higher BAP rates than fathers (all *p’*s < 0.05), except for the self-report and informant-report Pragmatic language subscales, and the informant-report and best-estimate Rigidity subscales. Among parents of typically developing children, mothers had higher BAP rates than fathers (all *p’*s < 0.05), except for the self-report, informant-report, and best-estimate Rigidity subscales and the informant-report Pragmatic language subscale.

**Table 3:** Percentages of parents who met the criteria for BAP in the two parental groups

		Fathers						Mothers				
	Numbers in PCA	Percentages in PCA (%)	Numbers in PTC	Percentages in PTC (%)	χ^2^	*p*-value	Numbers in PCA	Percentages in PCA (%)	Numbers in PTC	Percentages in PTC (%)	χ^2^	*p*-value
Self-report BAPQ												
Aloof personality	29	9.7	11	4	7.1	0.008	60	20.1	35	12.8	5.5	0.019
Pragmatic language	56	18.7	23	8.4	12.8	<0.001	75	25.1	55	20.1	2	0.153
Rigidity personality	21	7	13	4.7	1.3	0.249	39	13	18	6.6	6.7	0.01
Total score	27	9	10	3.6	6.9	0.009	87	29.1	48	17.5	10.6	0.001
Informant-report BAPQ												
Aloof personality	18	6	10	3.6	1.7	0.189	57	19.1	31	11.3	6.6	0.01
Pragmatic language	59	19.7	28	10.2	10	0.002	76	25.4	43	15.7	8.2	0.004
Rigidity personality	26	8.7	9	3.3	7.3	0.007	22	7.4	13	4.7	1.7	0.192
Total score	33	11	13	4.7	7.7	0.006	64	21.4	28	10.2	13.3	<0.001
Best-estimate BAPQ												
Aloof personality	24	8	6	2.2	9.8	0.002	63	21.1	35	12.8	6.9	0.008
Pragmatic language	63	21.1	27	9.9	13.6	<0.001	97	32.4	60	21.9	8	0.005
Rigidity personality	19	6.4	8	2.9	3.8	0.053	23	7.7	12	4.4	2.7	0.098
Total score	39	13	8	2.9	19.5	<0.001	92	30.8	57	20.8	7.4	0.007

BAP: Broad Autism Phenotype; BAPQ: Broad Autism Phenotype Questionnaire; PCA: parents of children with autism; PTC: parents of typically developing children.

### Co-occurrence of BAP features

**Fig. 1:**
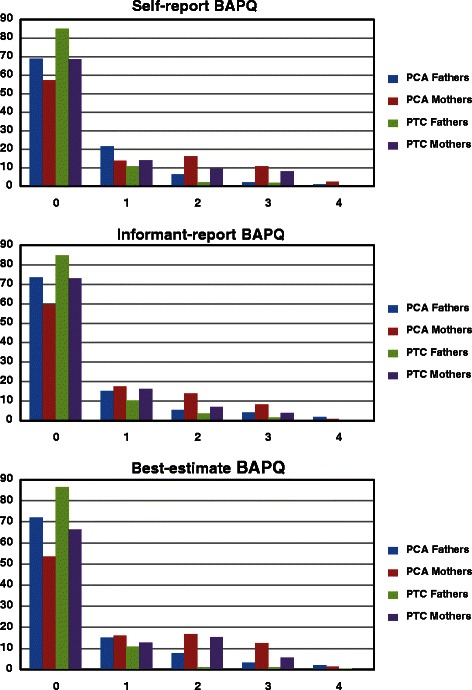
Proportion of PCA and PTC fathers and mothers with 0, 1, 2, 3, and 4 BAP features on the three versions of the BAPQ. BAP: Broad Autism Phenotype; BAPQ: Broad Autism Phenotype Questionnaire; PCA: parents of children with autism; PTC: parents of typically developing children

Co-occurrence was examined by comparing the percentage of parents with multiple BAP features for the three versions of the BAPQ in the two parent groups (i.e., parents of children with autism and parents of typically developing children). Figure 1 shows the proportion of fathers and mothers in the autism group and the typical development group with 0 to 4 BAP features. As indicated in Fig. 1, 9.4 % of the fathers of children with autism had more than one BAP feature compared with 4.0 % of fathers of typically developing children (χ2 = 6.452, *p* = 0.011) on the self-report BAPQ. Moreover, 29.10 % of mothers of children with autism had more than one BAP feature in contrast to 17.5 % of mothers of typically developing children (χ2 = 10.644, *p* = 0.001). The parents (fathers and mothers) of children with autism also had higher rates of co-occurrence of BAP features on the other versions of the BAPQ, than parents of typically developing children (all *p’*s < 0.05).

### Relationship between parental BAPQ scores and children SRS total scores

Sixty-three children with autism and 63 typically developing children who were younger than four years old were excluded from this analysis because the SRS is not applicable for children under four years of age. There was no significant difference in age between the autistic (M = 64.59 months, SD = 12.46) and the typically developing children (M = 64.08 months, SD = 12.65). Children with autism had significantly higher SRS total scores (M = 98.93, SD = 25.35) than typically developing children (M = 43.23, SD = 11.29; *t* = −30.532, *p* < 0.001).

Regression analyses were conducted to examine the relationship between each BAPQ total score of each parent and their child’s SRS total score. To check whether outliers were exerting influence on the regression results, the statistics such as residuals, leverage, and Cook’s D were used. We found out that there were 21 outliers in autism group and 21 outliers in control group on self- and informant-report BAPQ. There were 23 outliers in autism group and 21 outliers in control group on best-estimate BAPQ. These outliers were excluded from this regression analysis. The results showed that fathers’ and mothers’ BAPQ total scores were significantly associated with their children’s SRS total scores (Self-report BAPQ ─ fathers: Beta = 0.157, *p* = 0.002; mothers: Beta = 0.194, *p* < 0.001; Informant-report BAPQ ─ fathers: Beta = 0.212, *p* < 0.001; mothers: Beta = 0.089, *p* = 0.084; Best-estimate BAPQ ─ fathers: Beta = 0.173, *p* = 0.004; mothers: Beta = 0.16, *p* = 0.007). To investigate the differential effects of paternal and maternal BAPQ total scores on their child’s social functioning, separate regression models were conducted for the autistic and control groups. We found that the three versions of BAPQ total scores of the mothers of children with autism were not significantly associated with their children’s SRS total scores (see Table 4), whereas the three versions of BAPQ total scores of the mothers of typically developing children were significantly associated with their children’s SRS total scores. The informant- and best-estimate BAPQ total scores of the fathers of children with autism were significantly associated with their children’s SRS total scores, whereas the self- and best-estimate BAPQ total scores of the fathers of typically developing children were not significantly associated with their children’s SRS total scores.

**Table 4:** Results from regression analysis predicting children’s SRS total scores from parental BAPQ total scores

	Autism				TDC			
	Beta	*t*	*p*	Number	Beta	*t*	*p*	Number
Mother’s self-report BAPQ total scores	136	1.819	0.07	215	0.38	5.418	0.00	190
Father’s self-report BAPQ total scores	0.096	1.282	0.201	215	−0.046	−0.65	0.517	190
Mother’s informant-report BAPQ total scores	−0.032	−0.464	0.643	215	0.164	2.077	0.039	190
Father’s informant-report BAPQ total scores	0.22	3.179	0.002	215	0.196	2.477	0.014	190
Mother’s best-estimate BAPQ total scores	0.073	0.9	0.369	213	0.34	3.854	0.00	190
Father’s best-estimate BAPQ total scores	0.16	1.986	0.048	213	0.045	0.513	0.609	190

BAPQ: Broad Autism Phenotype Questionnaire; SRS: Social Responsiveness Scale; TDC: typically developing children.

Parents were classified as “BAP present” if at least one parent met the criteria for BAP in at least one domain (Aloofness personality, Pragmatic language, Rigidity, or total score). Independent samples t-tests revealed that, in the autism group, children of“BAP present” parents had higher SRS total scores for the informant-report (*p* = 0.034) and best-estimate BAPQ (*p* = 0.004) than children of“BAP absent” parents. In the typical development group, children with“BAP present” parents had higher SRS total scores for best-estimate BAPQ (*p* = 0.011) than children with “BAP absent” parents (Table 5).

**Table 5:** Mean SRS total scores in BAP absent and BAP present parents: autism and typically-developing children

Group	BAPQ version	BAP absent number	SRS BAP absent	BAP present number	SRS BAP present	t	*p*
Autism	Self-report	103	95.49 (24.71)	133	101.59 (25.62)	−1.845	0.066
	Informant-report	113	95.27 (24.17)	123	102.28 (26.04)	−2.138	0.034
	Best-estimate	105	93.66 (24.69)	131	103.15 (25.18)	−2.904	0.004
TDC	Self-report	128	42.16 (11.08)	83	44.89 (11.48)	−1.727	0.086
	Informant-report	141	42.62 (10.94)	70	44.47 (11.95)	−1.124	0.262
	Best-estimate	138	41.80 (11.31)	73	45.93 (10.83)	−2.559	0.011

TDC: typically developing children; BAP: broad autism phenotype; BAPQ: Broad Autism Phenotype Questionnaire; SRS: Social Responsiveness Scale.

## Discussion

Both parents of children with autism in the present study had significantly higher total scores on all three versions of the BAPQ than parents of typically developing children. They also scored significantly higher on the Aloof personality and Pragmatic language subscales than parents of typically developing children. These findings are generally consistent with the earlier findings of Sasson and colleagues [13], which showed that PCA mothers and fathers had higher best-estimate scores on all the BAPQ subscales than control parents had. The current findings regarding sex differences in BAPQ scores did not support prior findings, which reported that the fathers of children with autism and males, in general, scored higher than females on BAP features [7, 14, 21–24]. Mothers in the current study had significantly higher scores than fathers on the informant-report Pragmatic language and the Rigidity subscales, and total score, as well as on the best-estimate Rigidity subscale. However, the mothers and fathers of children with autism did not differ significantly on any of their BAPQ scores. Robel et al. [25], who used a French version of the Autism Spectrum Quotient (AQ) to study French samples of parents of children with autism also failed to find sex differences between parents. Further research using another measure of BAP with a sample of Chinese parents of children with autism is needed to confirm this finding.

The percentages on parents with BAP features on each subscale and version of the BAPQ in our sample ranged from 7 % to 32 % among mothers of children with autism and from 4 % to 22 % among mothers of typically developing children. By comparison, Western studies have reported that 10 %-23 % [13] and 10 %-12 % [15] of mothers of children with autism have BAP features and 4 %-10 % [13], 1 %-6 % [15] of mothers of typically developing children have BAP features. The rate of BAP features ranged between 6 % and 21 % among fathers of children with autism and between 2 % and 10 % among fathers of children with typical development. These rates are generally consistent with Western studies, which have reported that roughly 13 %-21.6 % of fathers of children with autism and 4.7 %-12.4 % of fathers of typically developing children met the criteria for BAP [13, 15]. Mothers had higher BAP rates than fathers in our PCA group, which is inconsistent with Western studies, which have reported that a larger percentage of fathers than mothers met the criteria for BAP [15, 17, 22, 26]. It is not known why the Chinese mothers have higher BAP rates, and further study is needed to determine whether cultural background leads to such discrepant rates, or whether the variation in rates are within the expected range of these BAP characteristics. The present study found parents of children with autism were more likely than parents of typically developing children to have more than one BAP feature. This finding is consistent with previous studies that reported biological parents of children with autism exhibit more BAP traits than other parents [13].

The BAP features of parents were associated with their child’s social functioning among all the study participants, but the patterns were different in the two groups of families. In the autism group, the informant-report and best-estimate BAP features of fathers were associated with their child’s social functioning, but a similar association was not found for mothers. In contrast, an association was found in the typically developing children group between the BAP features of the mothers on the three BAPQ versions and their child’s social functioning, but there was no association between the fathers’ BAP features on self-report and best-estimate BAPQ and their child’s social functioning. This finding is consistent with previous Western studies [15, 27], which found the BAP traits of fathers were associated more strongly with their autistic children’s social functioning than were the BAP traits of mothers. Autism has a strong genetic component, as indicated by the recurrence risk in families and in twin studies [28]. Previous studies have demonstrated that paternal and maternal genes are differentially expressed in offspring [29], and the differential expression is mediated by epigenetic modification [30, 31]. For example, the relative over-expression of paternal genes may drive cognition and behavior to “more demanding” phenotypes [32], as observed in Asperger syndrome [33]. Furthermore, some parent-of- origin genetic variants, such as some inherited copy-number variants [34, 35], are likely to contribute to the etiology of autism and, therefore, to lead to different effects on the phenotype of autistic children. Our findings provide evidence that the fathers of children with autism have an important influence on the severity of their child’s autistic symptoms, and suggest that the genetic transmission is different in families with and without autism. In the autism group, paternal BAP features were associated with the child’s social functioning in the informant-report and best-estimate BAPQ versions, but not in the self-report BAPQ. This may due to the fact that some fathers of children with autism lack insight into their BAP characteristics. In the typically developing children group, paternal BAP features were associated with the child’s social functioning only in the informant-report BAPQ versions, but not in the self-report and best-estimate BAPQ, which highlights the importance of using best-estimate BAPQ versions to measure parental BAP features.

Several limitations of this study should be considered when interpreting the results reported here. First, although the diagnosis of autism in the children in the study was made according to the DSM-IV, it was not confirmed by the Autism Diagnostic Observation Schedule (ADOS) [36] or the Autism Diagnostic Interview-Revised (ADI-R) [37], because these two standard diagnostic tools are not commonly used in China at present. Second, we did not obtain other important information, such as the children’s educational status and cognitive ability, or the parents’ mental and physical health status, which may affect children’s SRS scores and parental BAPQ scores. For example, previous research has reported that parents of children with autism had greater stress and depression and a lower quality of life than parents of children with other developmental disabilities [38–40]. Thus, mental health status could affect the response of the parents of children with autism. Third, the total raw scores on the SRS, which usually should be converted to gender-based T-scores, were used directly for comparison in this study in the absence of Chinese norms. Fourth, we only used the BAPQ to measure the BAP features of parents, and a study conducted with another BAP measurement questionnaire is required to validate our results.

## Conclusions

The current study increases our understanding of BAP and the relationship between parental BAP and children’s social functioning in a different cultural context than that of most prior research. The most striking aspects of our work are that: (1) parents of children with autism had higher BAP rates than parents of typically developing children, and that mothers had higher BAP rates than fathers in both parental groups; and (2) the patterns of association between parental BAP and children’s social impairment in autistic families is different from the pattern in families of children with typical development. The findings of our study have important implications for future genetic studies of autism, and may be applicable to some other neurodevelopmental and psychiatric disorders.

## Abbreviations

BAP: Broad autism phenotype
BAPQ: Broad Autism Phenotype Questionnaire
SRS: Social Responsiveness Scale
SCQ: Social Communication Questionnaire
DSM-IV: The Fourth Edition of the Diagnostic and Statistical Manual of Mental Disorders
